# Burden of metabolic syndrome on primary healthcare costs among older adults: A cross-sectional study

**DOI:** 10.1590/1516-3180.2023.0215.R1.13052024

**Published:** 2024-08-12

**Authors:** Suelen Jane Ricardo, Monique Yndawe Castanho Araujo, Lionai Lima dos Santos, Marcelo Romanzini, Rômulo Araújo Fernandes, Bruna C. Turi-Lynch, Jamile Sanches Codogno

**Affiliations:** I Student, Laboratory of Investigation in Exercise (LIVE), Department of Physical Education, Universidade Estadual Paulista (UNESP), Presidente Prudente (SP), Brazil.; II Postdoctoral student, Laboratory of Investigation in Exercise (LIVE), Department of Physical Education, Universidade Estadual Paulista (UNESP), Presidente Prudente (SP), Brazil.; III Student, Laboratory of Investigation in Exercise (LIVE), Department of Physical Education, Universidade Estadual Paulista (UNESP), Presidente Prudente (SP), Brazil.; IV Assistant Professor, Department of Physical Education, Universidade Estadual de Londrina (UEL), Londrina (PR), Brazil.; V Assistant Professor, Laboratory of Investigation in Exercise (LIVE), Department of Physical Education, Universidade Estadual Paulista (UNESP), Presidente Prudente, (SP), Brazil.; VI Assistant Professor, Department of Physical Education & Exercise Science, Lander University, Greenwood, USA.; VII Assistant Professor, Laboratory of Investigation in Exercise (LIVE), Department of Physical Education, Universidade Estadual Paulista (UNESP), Presidente Prudente (SP), Brazil.

**Keywords:** Hypertension, Health services research, Diabetes mellitus, Obesity, Exercise, National Health Service, Health economics, Physical activity

## Abstract

**BACKGROUND:**

The impact of metabolic syndrome (MetS) on healthcare costs remains unclear in the literature.

**OBJECTIVES:**

To determine the impact of MetS on primary healthcare costs of adults, as well as to identify the impact of physical activity and other covariates on this phenomenon.

**DESIGN AND SETTING:**

This cross-sectional study was conducted in the city of Presidente Prudente, State of São Paulo/Brazil, in 2016.

**METHODS:**

The sample comprised 159 older adults (> 50 years) of both sexes (110 women) who were identified from their medical records in the Brazilian National Health Service. Healthcare costs (US$) were assessed through medical records and divided into medical consultations, medications, laboratory tests, and total costs. MetS was assessed using medical records.

**RESULTS:**

The Brazilian National Health Service spent more on consultations (US$ 22.75 versus US$ 19.39; + 17.3%) and medication (US$ 19.65 versus US$ 8.32; + 136.1%) among adults with MetS than among those without MetS, but the costs for laboratory tests were similar (P = 0.343). Total costs were 53.9% higher in adults with MetS than in those without the diagnosis of the disease (P = 0.001). Regarding total costs, there was an increase of US$ 38.97 when five components of MetS were present (P = 0.015), representing an increase of approximately 700%, even after adjusting for sex, age, and physical activity.

**CONCLUSION:**

In conclusion, the presence of the MetS is responsible for increasing primary care costs among older adults, especially in those related to medicines.

## INTRODUCTION

Metabolic syndrome (MetS) is a complex disorder characterized by a set of cardiovascular risk factors.^1–4^ According to the National Cholesterol Education Program's Adult Treatment Panel III,^1^ the diagnosis of MetS is made by altering at least three of these components: abdominal obesity, high triglycerides (TG), low high-density lipoprotein (HDL), high blood pressure (BP), and high fasting blood glucose levels.^1^

Diabetes mellitus (DM), arterial hypertension (AH), and cardiovascular diseases (CVDs), in isolation, are known as public health problems responsible for high mortality rates worldwide.^1–5^ There are indications that MetS, in most cases, foreshadows some CVDs,^6–8^ which are responsible for the highest mortality rate (31% worldwide) according to the World Health Organization.^4^ People with MetS are more likely to have CVD than those without MetS (45.6% versus 8.6%, P < 0.0001) and show increased mortality risk of CVD.^7^ Another concern related to MetS is its increasing prevalence worldwide,^9–11^ which is a concerning phenomenon observed in both developed (Korea and United States)^9,10^ and developing nations (Brazil and India).^3,11^

In fact, the components of MetS contribute negatively to the economy.^11,12^ In the United States, patients with hypertension have generated an annual expenditure of approximately US$ 2,000 higher than people without the disease.^13^ In Brazil, a follow-up study identified that patients with DM spend approximately 41% more on health services when compared with individuals without DM.^12^ In contrast, the economic burden of MetS has been less investigated, mainly in developing nations.

Moreover, the literature confirms the positive impact of physical activity on the control of the different components of MetS^14^ and the mitigation of healthcare costs attributed to the treatment of diseases related to MetS,^15^ but its impact on the relationship between MetS and healthcare costs is not clear.

## OBJECTIVE

This study aimed to determine the impact of MetS on the primary healthcare costs of older adults from the Brazilian National Health System and to identify the impact of physical activity and other covariates on this phenomenon.

## METHODS

### Study Population

This cross-sectional study, conducted in March 2016, is the sixth data collection of a cohort study performed in the city of Presidente Prudente, Western State of São Paulo, Brazil (population estimated at ~ 200,000 inhabitants and human development index of 0.806).^16^ Data collection was conducted in two Basic Healthcare Units (BHUs) designated by the Municipality Administration (Municipal Health Department). BHUs are health facilities spread out in different areas of the city, offering primary health services (e.g., vaccination, consultations, medicine delivery) and representing the most distal arm of the Brazilian National Health System (maintained by the Federal Government, offering free of charge primary [BHU], secondary [hospital], and tertiary services [hospital] for more than 200 million citizens). This study was approved by the Research Ethics Committee of the Faculty of Science and Technology of the Universidade Estadual Paulista (UNESP) and Presidente Prudente Campus (CAAE: 13750313.2.0000.5402; date: 04/05/2013).

The sample size was based on the study by Boudreau et al.,^17^ which compared primary care costs according to the presence (US$ 813.00/year) and absence (US$ 625.00/year) of MetS (US$ 88.00 difference between groups). Thus, with an expected difference of US$ 88.00, statistical power of 80%, alpha error of 5%, and standard deviations (presence MetS US$ 197.00 and absence MetS US$ 171.00), the minimum sample size estimated for each group was 69 participants (138 total).

The inclusion criteria were: i) active registration at the BHU for at least 1 year, ii) age > 50 years, iii) participation in the previous five data collections, and iv) signing a written consent form.

### Dependent Variable

#### Direct costs with health services

The treatment costs for each patient in the BHUs were verified through medical records in a time horizon of 12 months prior to data collection.^18,19^

Information regarding the number and types of consultations, laboratory tests, and medications was obtained. Additionally, to calculate the consultation costs used at the BHU, costs of other services (e.g., attendance services, utility bills, and dispensed medication) were added.

Monetary values were estimated from the perspective of the SUS based on the sum of resources used directly in the treatment of the patient, and micro-costing (bottom up) approaches were used to estimate costs.^20^

The cost calculation for each health service was based on the methodology described below:^18,19,21,22^

- Medical consultations: Costs were retrieved from the SUS System Management Procedures Table (SIGTAP), provided by the Ministry of Health.
- Attendance services (e.g., scheduling, medication dispensing, and management): Costs were calculated using the daily salary rate of the professionals involved in the services provided (monthly salary divided by 30 d) and the average number of patients visiting daily (daily salary rate divided by the number of daily visits).
- Utility bills of the healthcare unit (electricity, water, and telephone): Costs were calculated using the average of the last 3 months for each utility bill divided by 30 d. The utility bill value was divided by the number of patients visiting daily.
- Medications dispensed, specific consumables, and diagnostic services (laboratory tests and others): Costs were calculated by multiplying the specific cost of each standard procedure by the number of procedures performed.
- Medication and laboratory test costs were calculated as the specific cost of each standard procedure multiplied by the number of procedures performed.

Information regarding salaries, costs of laboratory tests, medications, and utility bills was provided by the Municipal Secretary of Health. To convert this information into current values (R$), values referring to the year of the purchase were informed by the Municipal Health Department. Monetary values were updated in accordance with the official Brazilian inflation index (IPCA) and converted into US dollars (US$) using the official exchange rate on July 10, 2020, published by the Central Bank of Brazil.^23^

### Independent Variables

#### MetS Components

To check for the presence of MetS, we considered the following criteria:^1^ Levels of glucose, HDL, and TG were analyzed through blood collection after 12 h of fasting (performed and analyzed by a licensed laboratory that follows clinical standard guidelines, located in the city of Presidente Prudente). Cutoff point was established as glucose ≥ 110 mg/dL; TG ≥ 150 mg/dL; and HDL < 40 mg/dL for men or < 50 mg/dL for women. For central obesity, waist circumference (WC) was measured following the protocol of Lohman, Roche, Martorell (1988),^24^ with cutoff points of 102 cm for men and 88 cm for women.^1^ Systolic (SBP) and diastolic (DBP) blood pressure was assessed with the individual at rest, following the recommendations of the 7th Brazilian Guideline for Arterial Hypertension.^25^ The cutoff point was established as SBP ≥ 130 mmHg/DBP ≥ 85 mmHg.

Patients were classified into two groups: i) "presence of MetS" when three or more altered components were observed, and ii) "absence of MetS" when two or fewer altered components were observed.

### Covariates

Patients reported their age and sex. For statistical analyses, individuals were classified as under or over 65 years old. WC, SBP, and DBP were assessed by the research team. Nutritional status was assessed using the body mass index (BMI), calculated by dividing the weight (kg) by the square of the height (m). Overweight was considered when the BMI showed values between 25 and 29.9 kg/m² and obesity when BMI was ≥30 kg/m².^26^

Habitual physical activity (HPA) was verified through a questionnaire validated for Brazilian Portuguese,^27^ involving three components and 16 questions: occupational physical activity (questions 1 to 8), physical exercise practiced during leisure time (questions 9 to 12), and physical activity during leisure time and locomotion (questions 13 to 16). The answers were reported on a Likert scale, and the sum of the points was transformed into a score corresponding to the participants’ HPA.^27^ The possible HPA score ranged from 3 to 15. The higher the total score obtained with the sum of the three domains, the greater was the HPA level.

### Statistical analysis

Descriptive analyses included numerical and categorical data presented as median values and interquartile ranges. The Kolmogorov–Smirnov normality test was applied, and the Kruskal–Wallis and Mann–Whitney tests were used to detect differences between the two groups. Quantile regression was used to compare healthcare costs according to the number of MetS components, adjusting for covariates (differences are expressed as coefficients and their 95% confidence intervals [95% CI]). In this study, the independent variable (components of MetS) was composed of six groups (sum varied from 0 to 5, regardless of which component) to verify how each group of the independent variable affected the dependent variable (healthcare costs) in comparison with a reference group (0 components). The values of the dependent variables represent the 50th percentile. Statistical significance (P value) was pre-fixed at values below 5%, and software used was Stata 16.0 statistical software (StataCorp LLC, Texas, United States).

## RESULTS

The sample consisted of 159 patients, 110 women (69.2%) and 49 men (30.8%), with a mean age of 64.06 (8.65) years (74 participants met the criteria for "presence of MetS" [48%]).

Adults with MetS were older and heavier than those without the diagnosis of the disease (Table 1). Moreover, markers of obesity, abdominal obesity, and AH were poorly controlled in adults with MetS. Physical activity was similar across the groups (P = 0.638).

**Table 1:** Sample characteristics according to presence and absence of MetS (Presidente Prudente, 2018)

		Metabolic Syndrome		P value*
		Absence (n = 85)	Presence (n = 74)	
		Median (IR)	Median (IR)	
**Numerical Variables**				
	Age (Years)	61.28 (11.34)	65.38 (11.89)	**0.036**
	Weight (Kg)	67.10 (19.30)	74.60 (17.40)	**0.001**
	BMI (Kg/m²)	26.53 (5.50)	30.75 (7.00)	**0.001**
	WC (cm)	92.00 (15.10)	101.50 (14.30)	**0.001**
	SBP (mm/Hg)	123.00 (21.00)	134.50 (26.00)	**0.001**
	DBP (mm/Hg)	74.00 (16.00)	78.00 (12.00)	**0.031**
	HPA (score)	6.37 (1.80)	6.37 (1.90)	0.638
**Categorical Variables**		% (n)	% (n)	P value**
	AH	41.0 (32)	59.0 (46)	**0.003**
	Low HDL-c	30.7 (31)	69.3 (70)	**0.001**
	High TG	10.8 (7)	89.2 (58)	**0.001**
	High Glucose	19.5 (8)	80.5 (33)	**0.001**
	WC_(abdominal obesity)_	34.4 (33)	65.6 (63)	**0.001**

* Mann–Whitney test;

** Chi-square test;

IR = interquartile range; BMI = body mass index; WC = waist circumference; SBP = systolic blood pressure; DBP = diastolic blood pressure; AH = arterial hypertension; HPA = habitual physical activity; HDL-c = high density lipoprotein cholesterol; TG = triglycerides.

The overall costs of these adults were US$ 32,401.58, being 28% higher in adults with MetS (US$ 14,156.84 versus US$ 18,244.74). Brazilian National Health System spent more with consultations (US$ 22.75 versus US$ 19.39; + 17.3%) and medication (US$ 19.65 versus US$ 8.32; + 136.1%) among adults with MetS than among those without MetS; however, the costs for laboratory tests were similar (P = 0.343). Average total costs were 53.9% higher in adults with MetS than in those without the diagnosis of the disease (P = 0.001) (Table 2).

**Table 2:** Healthcare costs based on presence of metabolic syndrome (Presidente Prudente, 2018)

Health expenditures	Metabolic Syndrome		P value*
	Absence (n = 85)	Presence (n = 74)	
	Median (IR)	Median (IR)	
Consultation	19.39 (13.26)	22.75 (15.37)	**0.014**
Laboratory tests	0.0 (13.53)	0.0 (14.46)	0.343
Medication	8.32 (17.35)	19.65 (26.67)	**0.001**
Total	35.15 (31.91)	54.13 (48.25)	**0.001**

* Mann–Whitney's test;

IR = interquartile range; MetS = metabolic syndrome.

When the difference between costs was considered, in addition to the presence of MetS and sex, it was found that women with MetS spent more on the Brazilian National Health System with consultations (+ 34.6% than men and + 24.6% than other women without MetS), medications (+ 49.3% than men and + 67.6% than other women without MetS), and total (+ 249.8% than men and + 175.2% than other women without MetS) (Table 3). When the age of the individuals was considered, it was found that, in the presence of MetS, those aged < 65 and > 65 years spent more on healthcare than those aged < 65 years without MetS (best scenario) (Table 4).

**Table 3:** Healthcare costs according to sex and the presence of metabolic syndrome (Presidente Prudente, 2018)

Health expenditures	Metabolic Syndrome				P value*
	Absence		Presence		
	Men (n = 29)	Women (n = 56)	Men (n = 20)	Women (n = 54)	
	Median (IR)	Median (IR)	Median (IR)	Median (IR)	
Consultation	16.44 (12.71)	17.76 (10.87)	15.50 (12.23)	22.13 (13.85)^a^,^b^,^c^	**0.005**
Laboratory tests	0.0 (14.67)	0.0 (10.37)	0.0 (12.30)	0.0 (12.74)	0.441
Medication	6.04 (13.16)	7.68 (17.07)	12.30 (17.85)	21.13 (24.57)^a^,^b^	**0.001**
Total	34.27 (35.05)	30.53 (23.86)	36.02 (28.79)	51.17 (40.13)^a^,^b^	**0.001**

* Kruskal–Wallis's test;

IR = interquartile range; MetS = metabolic syndrome;

a = different from the group Absence and Men;

b = different from the group Absence and Women;

c = different from the group Presence and Men.

**Table 4:** Healthcare costs according to age and presence of metabolic syndrome (Presidente Prudente, 2018)

Health expenditures	Metabolic Syndrome				P value*
	Absence		Presence		
	< 65 years (n = 54)	> 65 years (n = 31)	< 65 years (n = 36)	> 65 years (n = 38)	
	Median (IR)	Median (IR)	Median (IR)	Median (IR)	
Consultation	15.93 (13.01)	18.66 (9.12)	21.46 (15.06)^a^	19.31 (13.13)^a^	**0.044**
Laboratory tests	0.0 (9.58)	0.0 (15.32)	0.0 (14.87)	0.0 (0.0)	0.087
Medication	5.84 (12.84)	12.01 (18.64)	18.39 (22.32)^a^,^b^	17.24 (26.55)^a^	**0.001**
Total	29.12 (21.95)	40.56 (27.56)^a^	48.93 (39.93)^a^	40.48 (38.06)^a^	**0.001**

* Kruskal–Wallis test;

IR = interquartile range; MetS = metabolic syndrome;

a = different from the group Absence and < 65 years;

b = different from the group Absence and > 65 years.

In the quantile regression analysis (Table 5), when three or more components of MetS were present, there was a significant increase in medication costs. In detail, there was an increase of US$ 14.74 when three components of the MetS were present (71%; P = 0.040), US$ 14.88 when four components were present (72%; P = 0.047), and US$ 28.25 when five components were present (220%; P = 0.02).

**Table 5:** Quantile regression for the association between the number of metabolic syndrome components and costs (Presidente Prudente, 2018)

		Medication (US$)		Consultation (US$)		Total (US$)	
		β (β _95%CI_)	P value	β (β _95%CI_)	P value	β (β _95%CI_)	P value
**MetS _components_**							
	0	Reference	---	Reference	---	Reference	---
	1	8.62 (-5.31; 22.55)	0.223	-3.39 (-15.58; 8.80)	0.584	4.88 (-19.77; 29.53)	0.696
	2	2.63 (-11.45;16.72)	0.712	-4.64 (-16.96;7.69)	0.458	-4.24 (-29.16; 20.69)	0.737
	3	14.74 (0.66; 28.82)	**0.040**	-0.41 (-12.73;11.92)	0.948	18.24 (-.68; 43.16)	0.150
	4	14.88 (0.18; 29.57)	**0.047**	-4.85 (-17.72; 8.01)	0.457	10.27 (-15.74; 36.28)	0.436
	5	28.25 (10.60; 45.90)	**0.002**	9.38 (-6.06; 24.83)	0.232	38.97 (7.74; 70.20)	**0.015**
**Covariates**							
	Sex	2.08 (-5.33; 9.49)	0.580	4.32 (-2.17; 10.81)	0.190	6.91 (-6.21; 20.03)	0.300
	Age	0.24 (-0.15; 0.64)	0.221	0.07 (-0.27; 0.42)	0.680	0.51 (-0.18;1.21)	0.148
	HPA	0.69 (-1.27; 2.65)	0.489	0.50 (-1.21; 2.22)	0.564	-0.16 (-3.63; 3.31)	0.929

MetS = Metabolic Syndrome; 95% CI = 95% confidence interval; US$ = USA dollar; HPA = habitual physical activity.

Regarding total costs, there was an increase of US$ 38.97 when five components of MetS were present (P = 0.015; compared to the presence of one component), representing an increase of approximately 700%, even after adjustments for sex, age, and HPA. However, when analyzing the effect of individual covariates on costs, there was no significant difference.

## DISCUSSION

This cross-sectional study explored the contributions of MetS components to primary healthcare costs among adults. Our findings suggest that three or more components of MetS, especially medications, have a significant impact on healthcare costs.

The presence of various components of MetS represents a concern to health systems due to its impact on patients’ health and economic burden.^3,13,18^ This concern is well-founded, as it is possible to observe an increase in the prevalence of MetS and its isolated components in some countries.^8,9,28^ In Brazil, MetS has shown high prevalence, as reported by Ramires et al. (2018).^11^ In our study, the prevalence of MetS was 48%, which is similar to another Brazilian study carried out in 2011 that estimated a prevalence of 53.7% in the population aged over 40 years.^29^ In fact, the prevalence of MetS seems to be a relevant public health concern, mainly because it represents the combination of other relevant cardiovascular and metabolic outcomes.

Our main finding was that individuals with MetS had higher costs of medication, consultation, and total costs than those without MetS, which corroborates the literature.^30^ Given that MetS is the sum of three or more components, and each component contributes individually and differently to the use of health services,^5,12,30^ it was expected that treatment for MetS would lead to significantly higher costs.^28^

There is strong evidence that type 2 DM, one of the components of MetS, even when isolated, leads to higher costs for health services (R$ 317.19 versus R$ 225.09) than in individuals without the disease.^12^ The same happens with AH, which was responsible for US$ 2,000.00 higher costs than non-hypertensive people,^13^ and obesity, showing twice as many costs (R$ 3,141.84 versus R$ 1,349.60) with hospitalization when compared to individuals with normal weight.^30^

Understanding the contribution of MetS components to universal health systems is of high importance Although our study did not find significant contributions of physical activity levels between the two MetS groups (presence/absence), Ramires et al.^11^ found that physical inactivity was present in 98.1% of Brazilian individuals diagnosed with MetS. Moreover, the beneficial impact of other manifestations of physical activity and exercise, such as yoga on MetS (and its costs), needs to be investigated in deep, such as yoga.^31^

Another important point is that our results, when stratified by sex and age, showed that female sex can be a potential factor in the increase in health spending and the presence of MetS. As shown in Table 4, the health spending in those aged < 65 and > 65 years were more expensive in the presence of MetS than in those without MetS. In contrast, Table 3 shows that in the presence of MetS, only women showed significant differences from those without MetS. Turi et al.^32^ aimed to evaluate the determinants of healthcare costs for patients receiving primary health care in the Brazilian National Health System and found that several factors influence increases in healthcare costs, such as physical inactivity and obesity. Among other factors, gender stands out for its association with different cost indicators, with women having higher healthcare costs for exams, medical consultations, and total expenses than men. A possible explanation for men's lower demand for primary health services could be related to differences in gender roles, which, according to social imagination, attributes care to the female sphere, requiring men to be associated with invulnerability, strength, and virility.^33^

This study has some limitations. First, our sample was selected in a BHU because of their medical records (not randomly), which may cause bias in costs due to high chances of being treated for other diseases not detected in this study. Furthermore, it was impossible to transform the scores provided by the HPA questionnaire into measures/units that could be used for exercise prescription. In addition, the cross-sectional design did not allow for cause-effect assumptions between costs and MetS. Moreover, our economic analysis focused on the primary level, and costs from the secondary and tertiary levels were not considered.

However, it is worth highlighting that this is one of the few studies conducted in developing nations to investigate the burden of MetS on primary care costs. Furthermore, the present findings are of great value, not only for public policy strategies, but also for health professionals involved in the prevention, control, and treatment of MetS components and, with the monetary information provided by the results of the present study, decision makers can estimate, in local contexts, the impact of the studied comorbidities on healthcare costs on primary care.

## CONCLUSION

In summary, MetS is responsible for increasing primary care costs among adults, particularly those related to medication. Our findings are particularly relevant for developing nations where the economic impact of MetS is an additional burden for the NHS in large populations, such as Brazil, India, and China.
